# Anomalous superconductivity in twisted MoTe_2_ nanojunctions

**DOI:** 10.1126/sciadv.adq5712

**Published:** 2025-01-29

**Authors:** Yanyu Jia, Tiancheng Song, Zhaoyi Joy Zheng, Guangming Cheng, Ayelet J. Uzan, Guo Yu, Yue Tang, Connor J. Pollak, Fang Yuan, Michael Onyszczak, Kenji Watanabe, Takashi Taniguchi, Shiming Lei, Nan Yao, Leslie M. Schoop, N. P. Ong, Sanfeng Wu

**Affiliations:** ^1^ Department of Physics, Princeton University, Princeton, NJ 08544, USA.; ^2^ Department of Electrical and Computer Engineering, Princeton University, Princeton, NJ 08544, USA.; ^3^ Princeton Materials Institute, Princeton University, Princeton, NJ 08544, USA.; ^4^ Department of Chemistry, Princeton University, Princeton, NJ 08544, USA.; ^5^ Research Center for Electronic and Optical Materials, National Institute for Materials Science, 1-1 Namiki, Tsukuba 305-0044, Japan.; ^6^ Research Center for Materials Nanoarchitectonics, National Institute for Materials Science, 1-1 Namiki, Tsukuba 305-0044, Japan.; ^7^ Department of Physics, The Hong Kong University of Science and Technology, Clear Water Bay, Kowloon 999077, Hong Kong, China.

## Abstract

Introducing superconductivity in topological materials can lead to innovative electronic phases and device functionalities. Here, we present a unique strategy for quantum engineering of superconducting junctions in moiré materials through direct, on-chip, and fully encapsulated 2D crystal growth. We achieve robust and designable superconductivity in Pd-metalized twisted bilayer molybdenum ditelluride (MoTe_2_) and observe anomalous superconducting effects in high-quality junctions across ~20 moiré cells. Unexpectedly, the junction develops enhanced, instead of weakened, superconducting behaviors, exhibiting fluctuations to a higher critical magnetic field compared to its adjacent Pd_7_MoTe_2_ superconductor. In addition, the critical current further exhibits a notable V-shaped minimum at zero magnetic field. These features are unexpected in conventional Josephson junctions and absent in junctions of natural bilayer MoTe_2_ created using the same approach. We discuss implications of these observations, including the possible formation of mixed even- and odd-parity superconductivity at the moiré junctions. Our results also demonstrate a pathway to engineer and investigate superconductivity in fractional Chern insulators.

## Teaser

Quantum engineering of moiré MoTe_2_ leads to unconventional superconducting phenomena.

## INTRODUCTION

Recent observations of fractional quantum anomalous Hall effect (*1*–*3*) in twisted bilayer molybdenum ditelluride (tMoTe_2_) (*4*–*6*) have confirmed the existence of fractional Chern insulators (FCIs) (*7*–*13*) in the absence of magnetic fields. The creation of superconductivity (SC) in FCIs can in principle lead to interesting electronic states of matter under unexplored experimental conditions (*14*, *15*). However, it is challenging to create SC using traditional means in such air-sensitive two-dimensional (2D) moiré materials. Here, we overcome such challenges by presenting a unique strategy for constructing high-quality superconducting junctions consisting of air-sensitive van der Waals (vdW) moiré materials, such as tMoTe_2_. We present systematic characterizations of the junction, including both the atomic structure and the electronic transport behaviors.

## RESULTS

### vdW-encapsulated 2D growth and nanojunctions

**Fig. 1.**
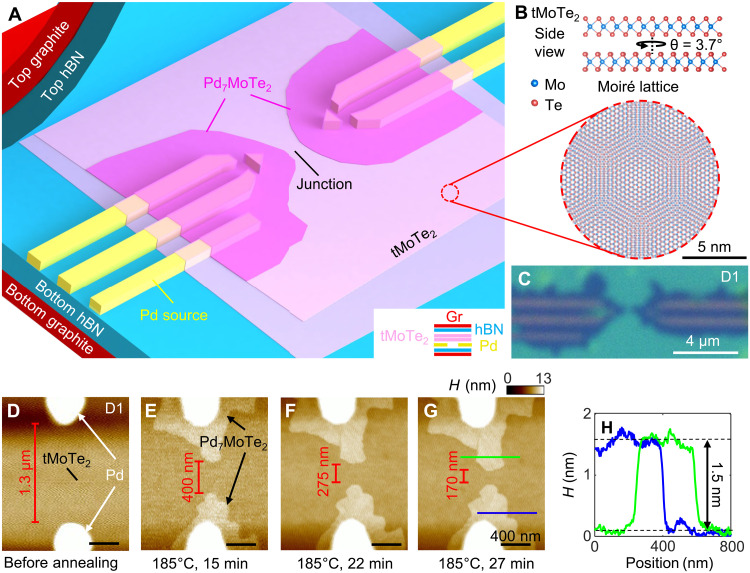
vdW-encapsulated, on-chip 2D growth on moiré materials and tMoTe_2_ junctions. (**A**) Cartoon illustration of the growth of Pd_7_MoTe_2_ superconductors and the device design. The cross-sectional device structure is illustrated in the inset at the bottom right. The junction including tMoTe_2_, Pd_7_MoTe_2_, as well as the Pd seed is fully encapsulated by the top and bottom graphite/hBN stacks. (**B**) tMoTe_2_ crystal lattice at an interlayer twist angle of ~3.7°. (**C**) Optical image of a typical device after on-chip growth. The darker regions extended from Pd contacts are thin Pd_7_MoTe_2_. (**D** to **G**) A series of AFM images of junction taken during a typical growth process with temperature and elapsed times is indicated below the images. The length of the tMoTe_2_ junctions is indicated by the red texts. (**H**) AFM height profiles along the solid lines indicated in (G) with matched color.

The key to our approach of fabricating high-quality superconducting moiré junctions is the recently introduced on-chip 2D growth mechanism (*16*), which is based on the unexpected discovery of a rapid mass transport and crystal growth templated on 2D materials. The air-sensitive 2H-MoTe_2_ flakes, in contact with predeposited palladium (Pd) source, are together fully encapsulated between top and bottom graphite/hexagonal boron nitride (hBN) stacks. The Pd serves as the seed of growth in the next step [see Fig. 1 (A and B) for cartoon illustrations of the device and the crystal structure]. The device as fabricated contains no superconducting materials (neither Pd nor MoTe_2_ superconducts). We then anneal the device at ~185°C, which triggers the transport of an ultrathin uniform layer of Pd into MoTe_2_ layer, and their reactions produce a new crystalline compound Pd_7_MoTe_2_, which can be seen as the darker region extending from the Pd contacts in the optical image of the final device after annealing (Fig. 1C). All processes involving MoTe_2_ are implemented in an Argon-filled glovebox to prevent degradation (Materials and Methods). Detailed characterizations of such a chamber-free 2D low-temperature synthesis, generalizable to various combinations of metals and 2D materials, can be found in (*16*). Using this approach, we find that a class of unique Pd-based compounds produced on topological chalcogenides, including the Pd_7_MoTe_2_ synthesized here, are superconductors (*17*).

Here, we demonstrate a controllable creation of high-quality superconducting junctions in bilayer MoTe_2_ in both its natural and twisted forms. Figure 1 (D to G) shows a typical growth of Pd_7_MoTe_2_ on tMoTe_2_ in device D1 in which we record the synthesis process under an atomic force microscope (AFM) after selected growth elapsed time. The length of the tMoTe_2_ junction between two Pd_7_MoTe_2_ islands can be accurately determined by controlling the growth time. The increased thickness in the vdW stack due to the Pd spread is ~1.5 nm (Fig. 1H), consistent with our previous report (*16*).

### Atomic characterization of the junction

**Fig. 2.**
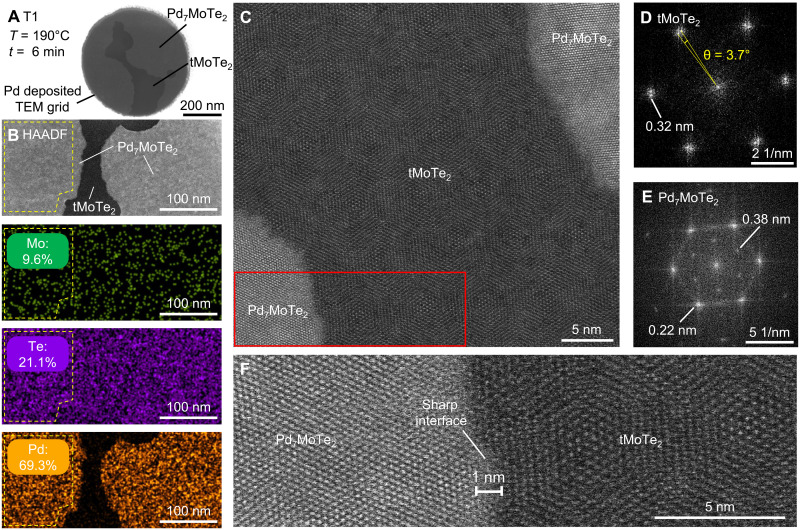
STEM analysis of tMoTe_2_ moiré junction with sharp interfaces. (**A**) STEM image of tMoTe_2_ after the growth of Pd_7_MoTe_2_, prepared on a TEM grid (sample T1). The TEM grid is predeposited with Pd, followed by transferring tMoTe_2_ on top. Subsequently, Pd is introduced by holding the temperature at 190°C for 6 min, resulting in a tMoTe_2_ moiré junction with a width of approximately 30 nm. Regions of different materials are indicated. (**B**) HAADF and corresponding elemental mappings captured at the moiré junction. The atomic ratio of Pd:Mo:Te is found to be close to 7:1:2 in the compound as grown, while negligible Pd can be observed in the neighboring tMoTe_2_ area. (**C**) An atomic-resolution STEM image of the moiré junction. (**D**) The FFT pattern of tMoTe_2_ regime, confirming the twist angle of 3.7°. (**E**) The FFT pattern of the crystalline Pd_7_MoTe_2_, showing the sixfold symmetry. (**F**) Magnification of the STEM image at the interface marked in the red rectangle in (C). The crystalline structures of both Pd_7_MoTe_2_ and tMoTe_2_ can be clearly visualized, demonstrating a sharp interface between the two regions. The moiré structure of tMoTe_2_ remains intact.

We first characterize the new compound Pd_7_MoTe_2_ and the nanojunction using a scanning transmission electron microscope (STEM), following the fabrication and experimental procedures described in (*16*, *18*) for both cross section and plan-view STEM studies. Figure 2A shows a STEM image of a suspended tMoTe_2_ film placed on a Pd-deposited TEM grid (sample T1), after the heat treatment at 190°C for 6 min. The lateral growth of the compound (brighter area) from the Pd outer seed leads to a junction of Pd_7_MoTe_2_/tMoTe_2_/ Pd_7_MoTe_2_ where the tMoTe_2_ gap is about 30 nm wide. We performed energy-dispersive x-ray (EDX) spectroscopy analysis on the film (Fig. 2B) and confirmed that the atomic ratio of Pd/Mo/Te in the compound is always very close to 7:1:2. Figure S1 shows EDX analysis on other locations that are well separated, confirming the uniformity of the as-grown material. Figure 2C displays high-angle annular dark-field (HAADF) images with an atomic resolution, which clearly reveals both a crystalline structure of the Pd_7_MoTe_2_ and the moiré lattice of tMoTe_2_. The fast Fourier transform (FFT) pattern of the region tMoTe_2_ (Fig. 2D) confirms its lattice structure as well as a twist angle of 3.7°, which is the target angle during fabrication. The FFT pattern of Pd_7_MoTe_2_, as shown in Fig. 2E, is indistinguishable from our previous observation (*16*) of Pd_7_WTe_2_, implying that the two crystal structures are the same. It is remarkable that an exceptionally sharp lateral interface (<1 nm) between Pd_7_MoTe_2_ and tMoTe_2_ is achieved and that the moiré structure of tMoTe_2_ remains intact in the junction (Fig. 2F). Figure S2 shows the cross-sectional STEM images taken from a Pd_7_MoTe_2_/tMoTe_2_ device (sample T2) grown inside a vdW stack fully encapsulated by hBN, like the transport device shown in Fig. 1. The data there again confirm the high quality of the junction.

We note that in regular devices with prepatterned contacts, the moiré structure near the contact region (within ~10 nm laterally) is typically distorted or even destroyed. The realization of a uniform moiré pattern extending to nearly atomically close to the metal contact in our experiment is outstanding. Our gentle and low-temperature growth helps improve and protect the moiré homogeneity because the ultrathin Pd film serves as a glue that seals the two MoTe_2_ monolayers, an advantage of our approach.

### Anomalous SC in tMoTe_2_ junctions

**Fig. 3.**
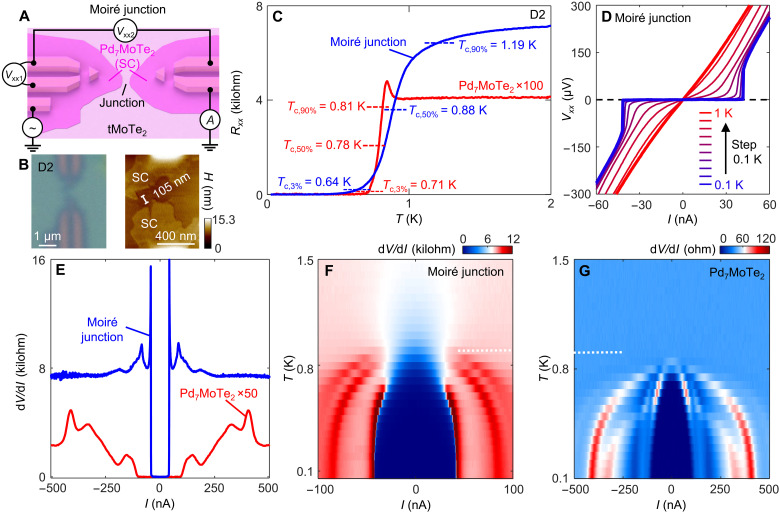
SC across the tMoTe_2_ moiré junction. (**A**) Cartoon illustration of transport measurement scheme. *V*_xx1_ and *V*_xx2_ are voltage drops recorded on Pd_7_MoTe_2_ and across the moiré junction, respectively. (**B**) Optical (left) and AFM (right) images of D2 (moiré junction; twist angle, ~3.7°). (**C**) Resistance as a function of *T*, for both Pd_7_MoTe_2_ (red) and the junction (blue). The critical temperatures, *T*_c,3%_, *T*_c,50%_, and *T*_c,90%_, are indicated. (**D**) *IV* characteristic curves for the moiré junction, taken at various *T* as indicated. (**E**) Differential resistance (d*V*/d*I*) versus an applied dc current (*I*) to the source, for both Pd_7_MoTe_2_ (red) and the junction (blue). (**F**) d*V*/d*I* versus *I* taken across the moiré junction under varying *T*. (**G**) The same d*V*/d*I* map for Pd_7_MoTe_2_ superconductor. *T* = 0.9 K is indicated by white dotted lines in both (F) and (G) as reference.

In the transport study of this work, we focus on SC properties of junctions with a width *d* ~ 100 nm, which we call short junctions. We demonstrate that SC is achieved across the junction of tMoTe_2_ in device D2, fabricated with an interlayer twist angle of ~3.7° and a junction *d* ~ 105 nm (across ~20 moiré cells) (Fig. 3, A and B). Figure 3A illustrates our transport measurement scheme that detects the voltage drops both on the Pd_7_MoTe_2_ and across the tMoTe_2_ junction when current passes through. The measured resistances (*R*_xx_) versus temperature (*T*) exhibit sharp decreases to zero just below ~1 K for both Pd_7_MoTe_2_ superconductor (*17*) and the tMoTe_2_ junction (Fig. 3C). The *IV* characteristics across the junction (Fig. 3D) display the expected SC nonlinearity and a sharp transition at a critical current (*I*_c_) of ~42 nA. The differential resistance d*V*/d*I* versus applied dc current (*I*), taken for both Pd_7_MoTe_2_ and the junction, reveals a smaller critical current across the junction (Fig. 3E).

However, we observe major differences between the moiré junctions and a conventional Josephson junction. The first hint of anomaly is that the SC fluctuations of the junction exist up to a higher temperature than Pd_7_MoTe_2_. As shown in Fig. 3C, we characterize the superconducting transition by presenting *T*_c,3%_, *T*_c,50%_, and *T*_c,90%_, defined as the temperatures at which *R*_xx_ drops to 3, 50, and 90% of the normal state value, respectively. Note that, in general, *T*_c,3%_ is close to the temperature at which the 2D superconductor loses its phase rigidity, whereas *T*_c,90%_ signifies the temperature up to which substantial superconducting fluctuations (hence pair strength) are still present. In our observations, while *T*_c,3%_ of the moiré junction is lower than that of Pd_7_MoTe_2_, *T*_c,90%_ of the moiré junction (~1.2 to 1.3 K) is, however, higher (*T*_c,90%_ of Pd_7_MoTe_2_ is ~0.8 to 0.9 K). We further highlight this feature in Fig. 3 (F and G), where d*V*/d*I* curves are recorded upon warming up the device. When Pd_7_MoTe_2_ is in its normal state (*T* > 0.9 K), substantial nonlinearity in *IV* curves remains across the junction at ~1.5 K.

**Fig. 4.**
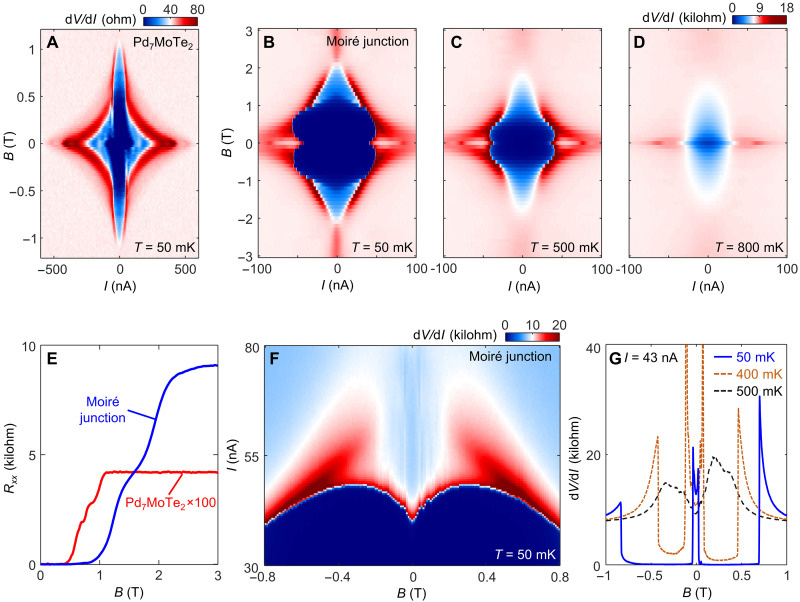
Anomalies of the superconducting moiré junction. (**A**) d*V*/d*I* versus *I* of the Pd_7_MoTe_2_ superconductor under varying magnetic field (*B*), taken at *T* = 50 mK. (**B**) The same map but for the moiré junction, at *T* = 50 mK. (**C** and **D**) The same map of the junction at *T* = 500 mK (C) and 800 mK (D), respectively. (**E**) Resistance as a function of *B*, for both Pd_7_MoTe_2_ (red) and the junction (blue). (**F**) d*V*/d*I* map taken under varying *B* and *I*, at 50 mK, highlighting the V-shaped critical current minimum at zero *B*. (**G**) d*V*/d*I* versus *B*, taken at a fixed dc current of 43 nA, under three different *T* as indicated.

The most notable features of the moiré junctions emerge when a magnetic field (*B*) is applied normally to the film. Figure 4 (A to D) displays the critical current behaviors of both Pd_7_MoTe_2_ and the moiré junction as a function of *B*. Whereas SC of Pd_7_MoTe_2_ at 50 mK is fully suppressed above ~1.2 T (Fig. 4A), features of SC at the junction persist to much higher fields (~2.2 T). The contrast is most apparent in Fig. 4E, where we compare their resistive transitions versus *B*. Both higher *T*_c_ and higher critical *B* (*B*_c_) suggest that the pairing potential in the junction is enhanced over that in the Pd_7_MoTe_2_ pads and that the usual proximity effects are not sufficient to explain our data. The data suggest that SC with a pairing potential distinct from that in Pd_7_MoTe_2_ appears to reside in the junction.

This is accompanied by another unexpected feature in the field profile of the critical current. In a conventional Josephson junction, winding of the supercurrent phase results in a pattern in which *I*_c_ peaks at *B* = 0. However, the moiré junctions exhibit a clear “V-shaped” minimum at zero *B*, as seen in Fig. 4 (B, C, and F). A different perspective of the anomalous minimum in *I*_c_ is seen if we plot d*V*/d*I* versus *B* with *I* fixed at 43 nA. We see that *B* drives the junction from a resistive state (at *B* = 0) to a dissipationless SC state as *B* increases (Fig. 4G). These anomalies, including the enhanced critical *B* and the V-shaped critical current minimum, are robust under repeated thermal cycling and highly reproducible across devices (see figs. S3 to S5 for data taken in another device, D3) and at all gate voltages (figs. S6 and S7).

An extrinsic cause of an enhanced *I*_c_ at finite *B* could be a quasiparticle-induced cooling effect, which has been discussed, for example, in the context of the nanowire junctions electrically contacted by bulk superconductor electrodes (*19*). These electrodes provide a cooling channel to the electrons in the junction. At finite magnetic fields, the creation of quasiparticles in the superconductor electrodes can provide better cooling for the junction electrons, potentially leading to higher *I*_c_ (lower electron temperature) of the junction in finite *B*. This electronic cooling is optimized when the superconducting electrodes become normal. However, our devices have a very different structure compared to the nanowire devices. Our electrodes are mainly made of Pd metal (nonsuperconducting), while the small area of superconducting Pd_7_MoTe_2_ is only located at the very tip of the electrodes. Also, the tMoTe_2_ is encapsulated within the graphite/thin hBN layers, where the graphite layers provide additional cooling power (the insulating hBN layer is only ~10 nm in thickness, offering poor thermal isolation). As a result, our device structure facilitates a good thermal equilibrium even at zero *B*. In the section below, we perform contrast experiments between natural bilayer and moiré bilayer junctions made using the same device geometry, components and fabrication process. The results, including the absence of the critical current anomaly in the natural bilayer junction, directly imply that this quasiparticle-assisted cooling mechanism cannot be essential in our devices.

A robust V-shaped minimum in *I*_c_ at zero *B* is quite rare. A well-known intrinsic example is the corner junction formed between a d-wave cuprate superconductor and an s-wave superconductor. In this situation, the destructive interference occurs between two spatially separated supercurrents with a relative phase shift of π (*20*). Likewise, destructive supercurrent interference can also be observed between spatially separated 0- and π-junctions in superconductor/ferromagnet/superconductor (SFS) junctions (*21*). In both cases, unconventional pairing and interference effects are key ingredients. We note an important distinction between our moiré junctions and SFS junctions. To see a V-shaped minimum in SFS junctions, one has to fine-tune the junction length *d* to subnanometer accuracy because the exchange splitting energy causes the sign of the Josephson coupling to oscillate rapidly with a spatial period of ~1 nm (*21*). By contrast, the V-shaped minimum here is observed in all devices without fine-tuning *d* (e.g., *d* ~ 105 nm in D2 and ~90 nm in D3). In addition, the time reversal symmetry is preserved in our junction. Guided by the corner-junction experiment on cuprate superconductors, we reason that the presence of destructive interference at the junction is needed for explaining the observed V-shaped minimum. Yet, the situation is also distinct from the cuprate corner junction since, in our case, the device is better described as a single junction without a corner geometry.

One possibility to reconcile all the experimental facts is to attribute such a destructive interference effect to the coexistence of an odd- and an even-parity condensate in the moiré junction. Both time reversal and inversion symmetries play key roles in the electron pairing of SC. In the presence of both symmetries, the conventional Bardeen-Cooper-Schrieffer theory favors an even-parity spin-singlet pairing. However, in the absence of either symmetry, unconventional pairing may occur. Particularly in materials with strong spin-orbit coupling (SOC) and broken inversion symmetry, SC with mixed even- and odd-parity states are anticipated (*22*–*24*). The possibility of unconventional pairing in noncentrosymmetric materials, e.g., heavy fermion systems, has been explored in the past decades (*23*–*25*), although challenges in confirming an odd-parity superconductor remain. In our devices, the SOC is quite large in tMoTe_2_, and the moiré lattice lacks inversion symmetry. Theoretically, this can lead to admixtures of even- and odd-parity pair condensates (*22*–*27*) in the moiré junction. We hence speculate that such a mixed pairing condensate may be responsible for generating two supercurrent channels with a π phase shift that destructively interfere. Whether the two channels are spatially overlapping or spontaneously separated awaits further experimental tests.

### Unusual normal state conduction in the junction

We further remark on the normal state resistance observed in short junctions (*d*, ~100 nm). MoTe_2_ is an insulator with a large activation gap (Δ, ~1 eV). However, in short junctions, we observe metallic behavior (even without applying a gate voltage) with a normal state resistance of several kilohms that is nearly *T* independent up to room temperature (Fig. 3C and figs. S3 and S8). The normal state resistance also exhibits little gate dependence (fig. S8). Below *T*_c_, these metallic-like junctions exhibit supercurrents as described. We note that the atomic resolution STEM studies of the junction (Fig. 2 and fig. S2) confirm the absence of Pd atoms in the tMoTe_2_ region in the junction, and the moiré structure remains unchanged. Although it is possible that some disorders are still present in the junctions, they are unlikely to be responsible for the residual conduction, which is highly consistent across different devices. Conduction due to tunneling events between rare hopping sites caused by disorders should strongly depend on temperature, in contrast to our experimental observations (fig. S8A).

One possible explanation for the unusual residual conduction is to assume that the band-bending effects close to the metallic Pd_7_MoTe_2_ pads could cause conduction channels in tMoTe_2_ junction to be populated. Although such an effect is expected at a metal-semiconductor interface, how it occurs in the ~100-nm bilayer MoTe_2_ junctions require careful consideration. As a comparison, we found that such residual conduction is absent in a monolayer MoTe_2_ junction with a width of ~65 nm fabricated using the same approach (see fig. S9). In addition, the induced electron density needed in the bilayer junctions seems quite high in this picture, since we cannot deplete it with electrostatic gating. The weak temperature dependence of the conductance in the normal state provides future constraints on its mechanism. We also suspect that the conduction may be via some hidden 1D electronic channels in the junction. Note that, for longer junctions with *d* > 500 nm, our experiments do show that the gate-tuned insulator state of tMoTe_2_ is recovered, following expectations. We now do not have a comprehensive explanation considering all the experimental facts. Future experiments, such as low-temperature scanning tunneling microscopy, would help resolve the situation.

Although the exact mechanisms for the normal state conduction and SC remain to be worked out, we next show that (i) the metallicity in these short junctions occur for both tMoTe_2_ and natural bilayer MoTe_2_, and yet (ii) only the tMoTe_2_ junction exhibits the superconducting anomalies. Namely, both the V-shaped minimum and the enhanced pair potential vanish if we perform the same experiments on the inversion symmetric natural bilayer.

### Contrasting behaviors in a natural bilayer junction

**Fig. 5.**
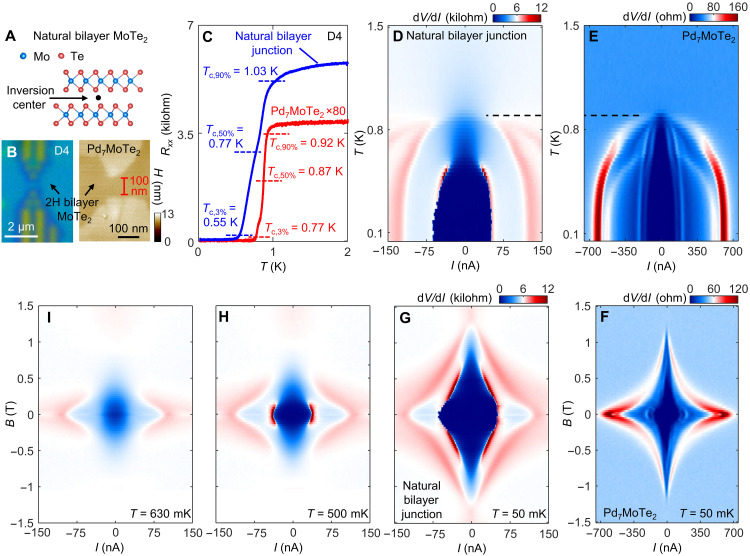
Absence of anomalies in the inversion symmetric bilayer junction. (**A**) Cartoon illustration of exfoliated natural bilayer 2H-MoTe_2_ lattice structure, where the inversion center is indicated. (**B**) An optical image (left) of a natural bilayer device after growth and a corresponding AFM image of the junction (right). (**C**) Resistance versus *T*, taken for Pd_7_MoTe_2_ (red) and the natural bilayer junction (blue) in this device. The same measurement geometry as Fig. 3A is used. The critical temperatures, *T*_c,3%_, *T*_c,50%_, and *T*_c,90%_, are indicated. (**D**) d*V*/d*I* map of the junction taken under varying *I* and *T*, showing the SC state. (**E**) The same map but for the Pd_7_MoTe_2_ SC. (**F**) d*V*/d*I* map of Pd_7_MoTe_2_, taken under varying *I* and *B*. *T* = 0.9 K is indicated by the black dotted lines in both (D) and (E) as reference. (**G** to **I**) The same d*V*/d*I* maps as (F) but taken for the junction, at three different *T* as indicated in the maps.

We now repeat the experiments described above on natural bilayer 2H-MoTe_2_, which hosts an inversion center located in the middle of the two layers (Fig. 5, A and B). The junction is fabricated using the same approach, with a similar length *d* ~ 100 nm. Both the unusual normal state conduction and the SC are consistently observed in the natural bilayer junction, yet the anomalous supercurrent features seen in tMoTe_2_ are now absent. Figure 5 (C to E) and fig. S10 display the *T*-dependent resistances of both the natural bilayer junction and the Pd_7_MoTe_2_ in the same device, similar to previous discussions. Characteristics of the Pd_7_MoTe_2_ superconducting pads in this device (D4) are closely similar to those in the moiré devices (D2 and D3), with similar values for the normal state resistance. The values of *I*_c_ when measured across the junctions are also similar. *T*_c_ of the junction is now slightly lower than the Pd_7_MoTe_2_ SC (Fig. 5C). The SC anomalies are now absent in the natural bilayer junction. (i) *B*_c_ of the junction also no longer exceeds that of Pd_7_MoTe_2_ (both ~1.2 T) (Fig. 5, F and G). Note that slight variance (±0.1 T) in *B*_c_ of Pd_7_MoTe_2_ found in different devices, especially between those grown on a natural bilayer and a moiré bilayer, could come from different levels of disorders and impurities in the compounds. Crucially, (ii) the V-shaped minimum in the *I*_c_ at zero *B* is now replaced by the conventional maximum as seen in Fig. 5 (G to I). The absence of the SC anomalies is confirmed at all gate voltages in the natural bilayer device (fig. S11). We further note that the anomalies we found here are also absent in Josephson junctions made of few layer WTe_2_ (*28*–*30*). We conclude that key properties of the tMoTe_2_ moiré junction, including the absence of inversion symmetry, are responsible for the superconducting anomalies.

## DISCUSSION

Future experimental and theoretical studies are necessary to uncover the underlying physics. While the understanding of the SC pairing symmetry certainly requires future experiments, the approach here based on moiré materials suggests a promising strategy to study unconventional pairing in noncentrosymmetric superconductors. The sharp interface between Pd_7_MoTe_2_ and tMoTe_2_ also implies a realistic route for engineering SC in moiré topological materials with the goal of proximitizing fractionalized states. Our current devices have a twist angle of ~3.7°, which has been shown to host the integer and FCI states upon electrostatic gating (*1*, *2*, *4*–*6*). Preliminary results in the current devices reveal a weak but interesting gate modulation of the junction critical current (fig. S12). Investigating the coexistence of SC and FCI states is possible with further optimization of the devices.

## MATERIALS AND METHODS

### Device fabrication

#### Transport devices (D1 to D4)

hBN and graphite flakes were exfoliated on SiO_2_/Si substrates, identified, and characterized under optical microscopes and AFM (Bruker Dimension Edge or Bruker Dimension Icon). Subsequently, hBN flakes were stacked on top of graphite flakes and then placed on SiO_2_/Si substrates. Electron beam lithography, followed by cold development, reactive ion etching and metal deposition, were used to create Pd contacts and growth seeds (~20-nm thick) on the bottom hBN/graphite stack. Before final assembly, the bottom stacks were then tip cleaned using AFM under the contact mode. To prepare the top stacks, we exfoliated monolayers and bilayers 2H-MoTe_2_ in an Ar-filled glovebox. For tMoTe_2_ devices (D1, D2, and D3), monolayer MoTe_2_ was cut into two pieces using a sharp tungsten tip. The first piece was picked up by the top vdW stack consisting of hBN and graphite flakes. The second piece of MoTe_2_ underwent a 3.7° rotation before being stacked with the first piece. For devices D4 and D5, natural bilayer and monolayer MoTe_2_ were directly picked up by a top hBN/graphite stack. The top stacks of MoTe_2_/hBN/graphite, for both types of devices, were then carefully aligned and positioned on the prepared bottom stacks. The devices prepared above were then AFM tip cleaned before being placed on a hot plate for the on-chip growth of Pd_7_MoTe_2_. With controlled temperature and time, the Pd growth process was carefully monitored under an optical microscope and an AFM. To achieve precise control, devices are initially monitored under an optical microscope until the two Pd_7_MoTe_2_ pads are approximately 500 nm apart. Then, according to the estimated growth rate, the devices are examined under AFM after each short-time extra growth to achieve the targeted junction length. The entire process involving MoTe_2_ was performed in a glovebox filled with argon, with concentrations of H_2_O < 0.1 parts per million (ppm) and O_2_ < 0.1 ppm.

#### TEM devices (T1 and T2)

The suspended TEM device (T1) was fabricated by stacking a 3.7° tMoTe_2_ onto a Pd-coated TEM grid using standard dry transfer technique. The polycarbonate (PC) used for the dry transfer was later removed by dissolving in chloroform for 30 min. Pd was introduced into the tMoTe_2_ by holding the TEM grid at 190°C for 6 min inside the vacuum chamber of STEM. For the TEM cross-sectional device (T2), the tMoTe_2_ stack and Pd_7_MoTe_2_ growth were created using the same process as for transport devices. A lamella specimen was then extracted from a selected region of the stack using a standard lift-out technique within a focused ion beam–scanning electron microscope system. The specimen was further thinned and polished using a Ga⁺ ion beam until it became sufficiently transparent for STEM analysis. All fabrication steps of both devices, including the removal of the PC layer, were conducted in an Ar-filled glovebox with H_2_O < 0.1 ppm and O_2_ < 0.1 ppm. More fabrication details can be found in (*16*–*18*).

### Transport measurements

The electrical transport measurement was conducted in a dilution refrigerator equipped with a superconducting magnet and a base temperature of ~20 mK. Four-probe resistance measurements were performed using the standard ac lock-in technique with a low frequency, typically ~23.3 Hz, and an ac current excitation from 0.5 to 10 nA. In addition, a dc current is also applied for critical current measurements.

### STEM measurements

Atomic resolution HAADF STEM imaging and EDX spectroscopy mappings were performed on a Titan Cubed Themis 300 double Cs-corrected STEM, equipped with an extreme field emission gun source and a super-X EDX system. The system was operated at 300 kV. A Gatan double tilt heating holder (model 652) was used for in situ heating study.
