# Supplementary material for: Anomalous superconductivity in twisted MoTe2 nanojunctions
Source: Sci Adv. 2025 Jan 29;11(5):eadq5712. doi: 10.1126/sciadv.adq5712 (PMC11777181; doi:10.1126/sciadv.adq5712)
Supplement: Supplementary file 1 — Figs. S1 to S12 [file sciadv.adq5712_sm.pdf]

Science Advances
AAAS

Supplementary Materials for

Anomalous superconductivity in twisted $MoTe_2$ nanojunctions

Yanyu Jia *et al.*

Corresponding author: Sanfeng Wu, sanfengw@princeton.edu; Yanyu Jia, yanyuj@princeton.edu

Sci. Adv. **11**, eadq5712 (2025)
DOI: 10.1126/sciadv.adq5712

This PDF file includes:

Figs. S1 to S12

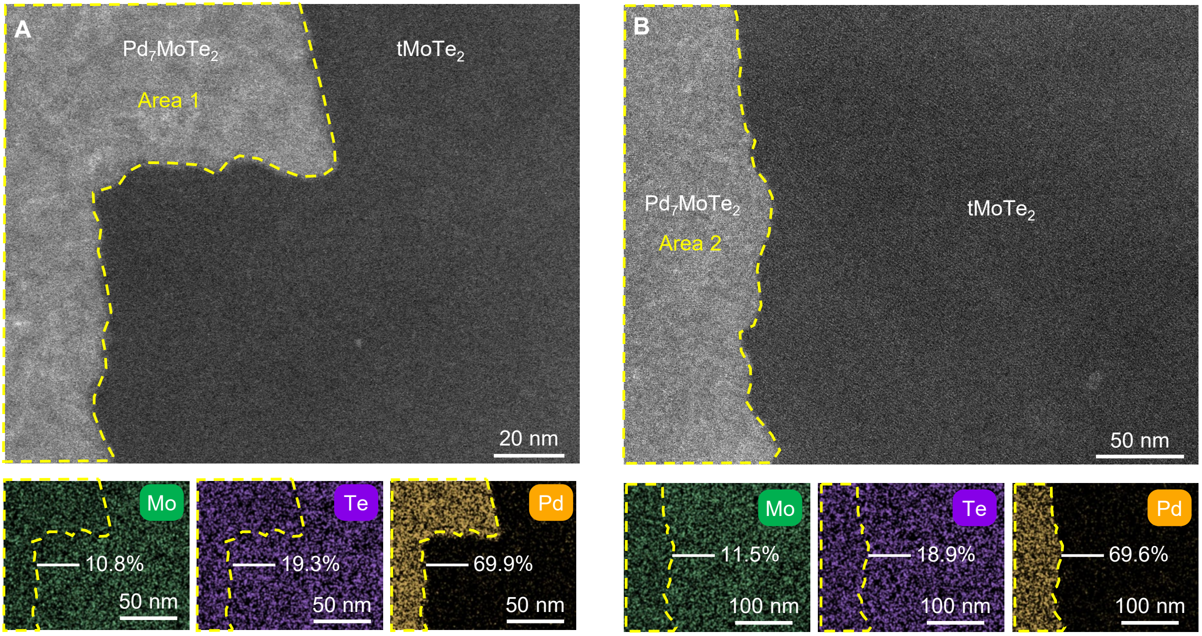

Fig. S1. Additional EDX analyses and elemental mappings (sample T1). A & B, The HAADF-STEM images (top) and corresponding EDX elemental mappings (bottom) captured at the interface between $tMoTe_2$ and Pd_7MoTe_2 at two additional well-separated locations of T1, show consistent results. The atomic ratios of Pd:Mo:Te in the new compound (outlined by the yellow dashed line) are always found to be approximately 7:1:2.

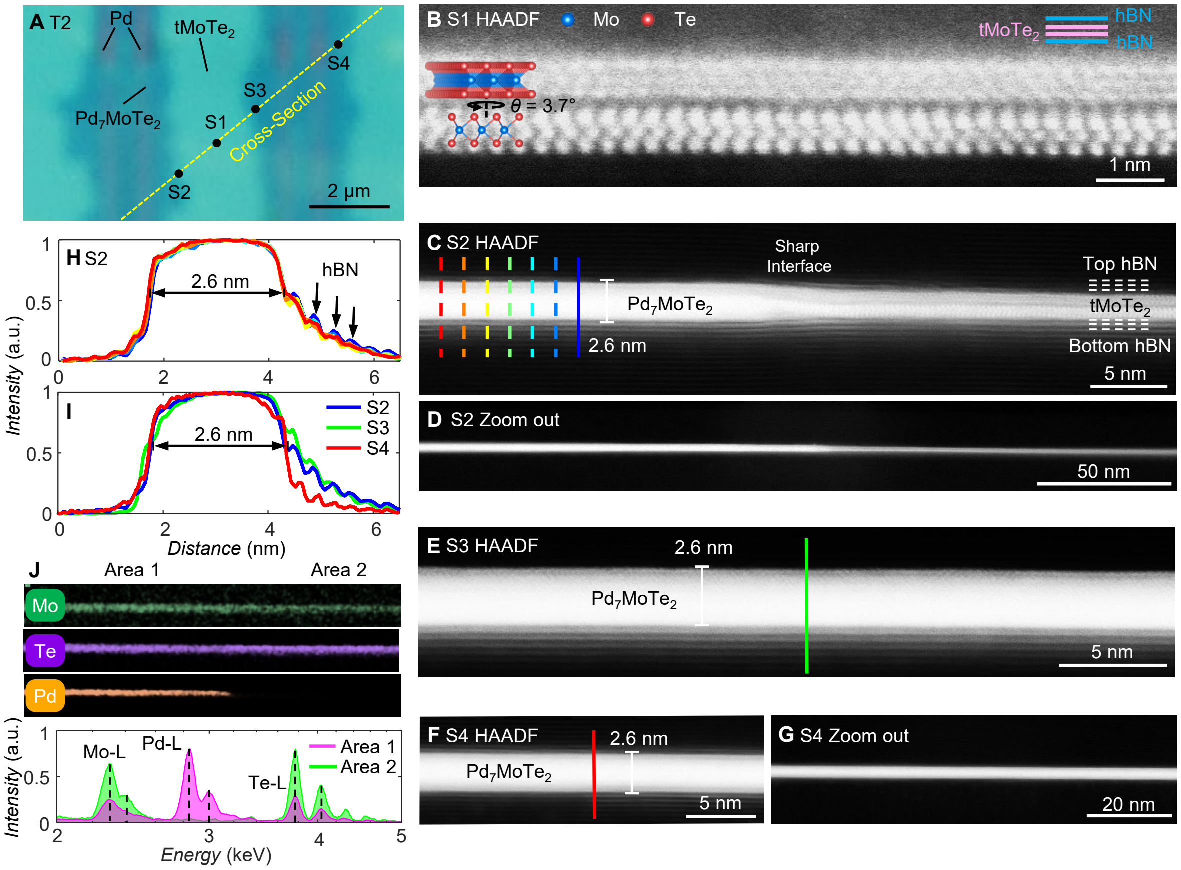

Fig. S2. Cross-section STEM studies of Pd_7MoTe_2 grown on $tMoTe_2$. A, Optical image of targeted region in sample T2, before being extracted for cross-section STEM studies. The STEM experiment was conducted to visualize the cross-section along the indicated yellow dashed line. Spots S1- S4 are estimated locations under examination in **B-G**. **B,** HAADF-STEM cross-section image of $tMoTe_2$ near spot S1. The atomic structure of bottom $MoTe_2$ layer is clearly resolved and the top layer appears blurry, reflecting the twist. **C,** HAADF-STEM cross-section image captured near S2, located at the boundary between Pd_7MoTe_2 and $tMoTe_2$. The thickness of Pd_7MoTe_2 is measured to be 2.6 nm. The extracted intensity along the colored line-cuts is shown in **H**. **D,** Zoomed-out STEM image near S2, illustrating the uniform thickness of Pd_7MoTe_2. **E-G,** The HAADF-STEM cross-section images taken near spots S3 and S4, located a few microns apart, confirming the same thickness and hence the uniformity of Pd_7MoTe_2. **H,** The extracted intensity along the line-cuts in **C**, indicated by the vertical lines. The signal from hBN layer structure is indicated by arrows. **I,** The extracted intensity from S2-S4 along line-cuts indicated by the solid lines in **C, E** & **F**, again verifying the uniform thickness of Pd_7MoTe_2 across the device. **J,** EDX elemental mappings and characteristic X-ray energy spectrum at the boundary between $tMoTe_2$ and Pd_7MoTe_2. Pd is only present in the Pd_7MoTe_2 area.

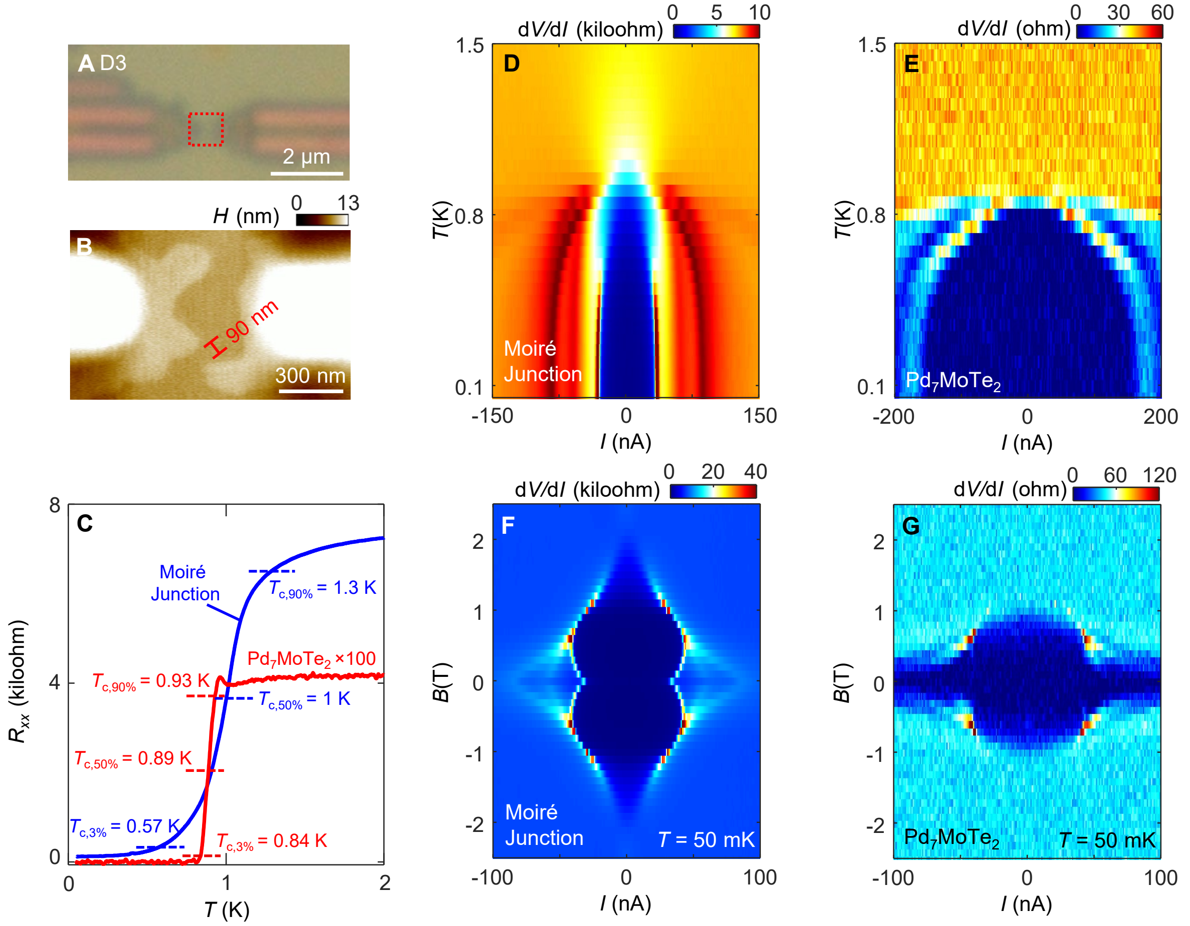

Fig. S3. Anomalous SC in another tMoTe₂ moiré junction (D3). A, Optical image of device D3 with a $tMoTe_2$ moiré junction (~ 3.7° twist angle) after Pd growth. **B,** AFM image of the moiré junction, revealing a junction length ~ 90 nm at the closest distance. The position of junction is highlighted in red box in **A**. **C,** Resistance *v.s.* T, taken for Pd_7MoTe_2 (red) and the moiré junction (blue) in this device. The same measurement geometry as Fig. 3A is used. The critical temperatures $T_{c,3\%}$, $T_{c,50\%}$ and $T_{c,90\%}$ are indicated. **D & E,** Temperature dependence of dV/dI *v.s.* I, taken across the moiré junction (**D**) and on Pd_7MoTe_2 (**E**), respectively. **F & G,** Magnetic field (B) dependence of dV/dI *v.s.* I, taken across the moiré junction (**F**) and on Pd_7MoTe_2 (**G**) respectively. Enhanced T_c and B_c and a V-shaped critical current minimum at zero B are robust, reproducible features.

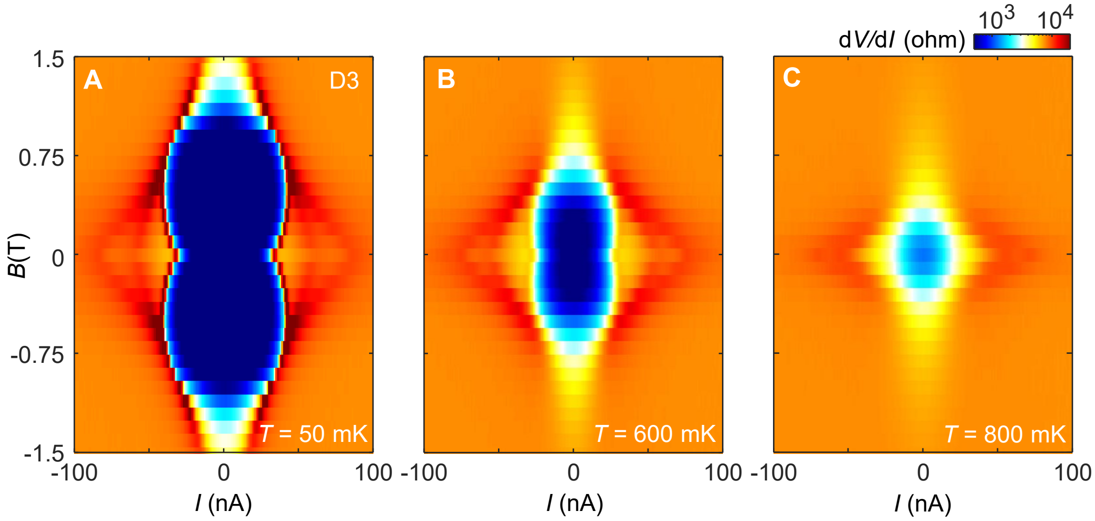

Fig. S4. Temperature dependence of the V-shaped critical current minimum in D3. A, The V-shaped critical current minimum observed in D3 at $T = 50$ mK. **B & C,** The same maps but taken at higher temperatures, $T = 600$ mK (**B**) and 800 mK (**C**), respectively.

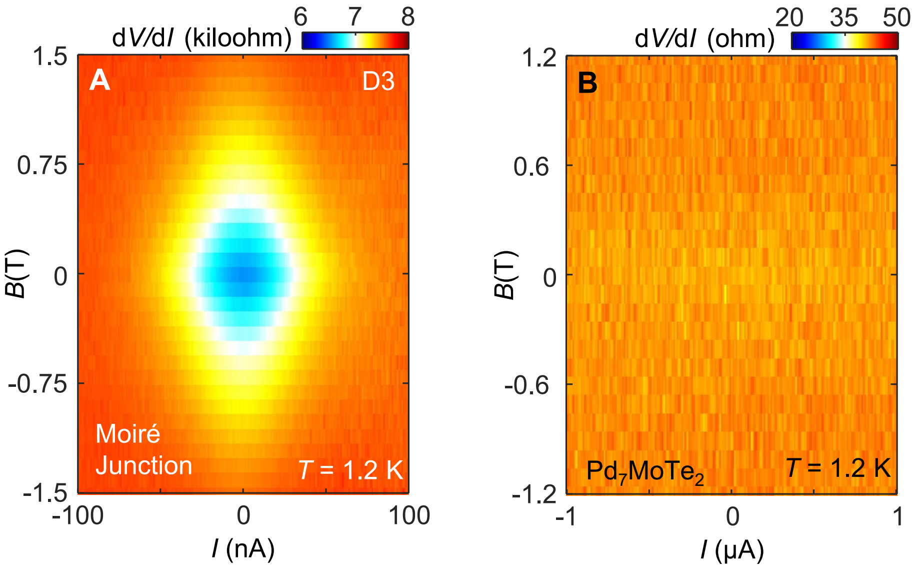

Fig. S5. Superconducting fluctuations at the moiré junction. A, dV/dI map of the moiré junction, as a function of B and applied DC current I, at T = 1.2 K, showing strong superconducting fluctuations. **B,** The same dV/dI map for Pd_7MoTe_2 taken in the same device, confirming that Pd_7MoTe_2 superconductivity is fully suppressed at this T.

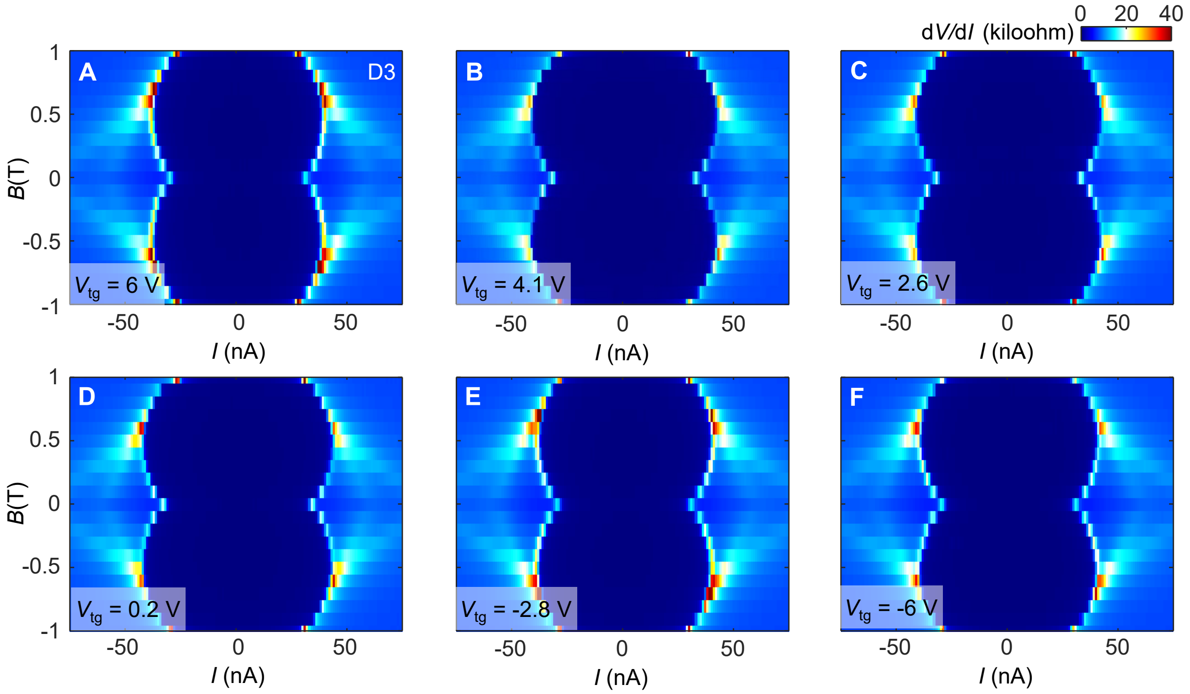

Fig. S6. Robust V-shaped critical current minimum taken at different gate voltages in D3. A-F, dV/dI(B, I) maps of the moiré junction in D3 under varying gates. The V-shaped critical current minimal is observed at all gate voltages (it is also robust against thermocycles).

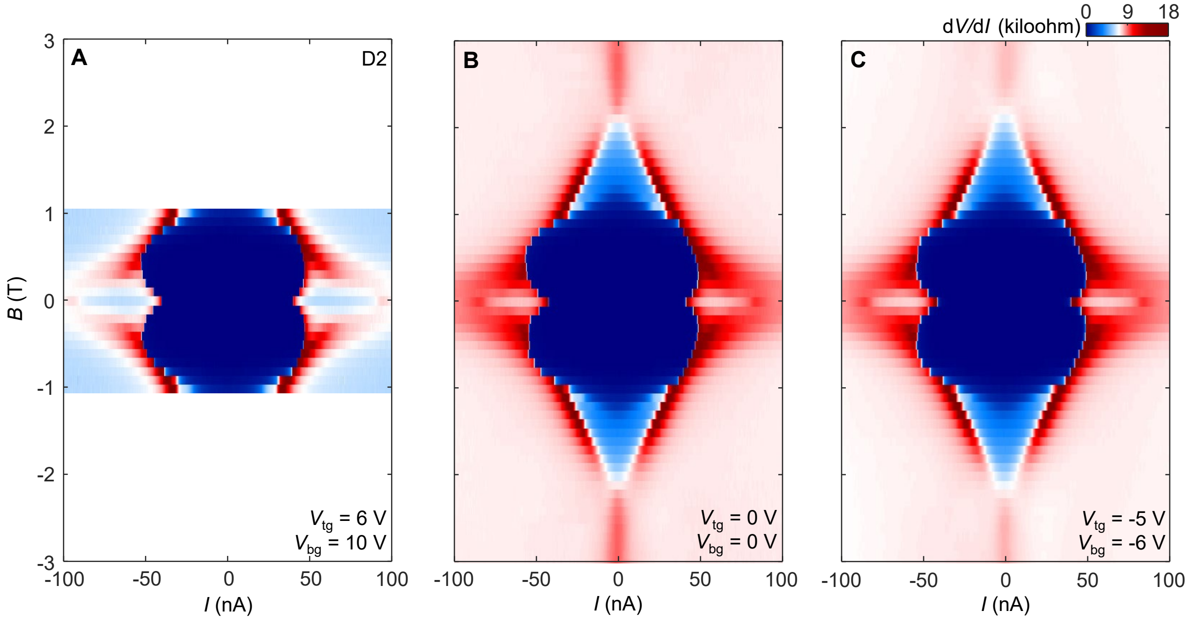

Fig. S7. Robust V-shaped critical current minimum taken at different gate voltages in D2. A-C, dV/dI(B, I) maps of the moiré junction in D2 under varying gate voltages and all displays the similar V-shaped critical current minimum at B = 0 T.

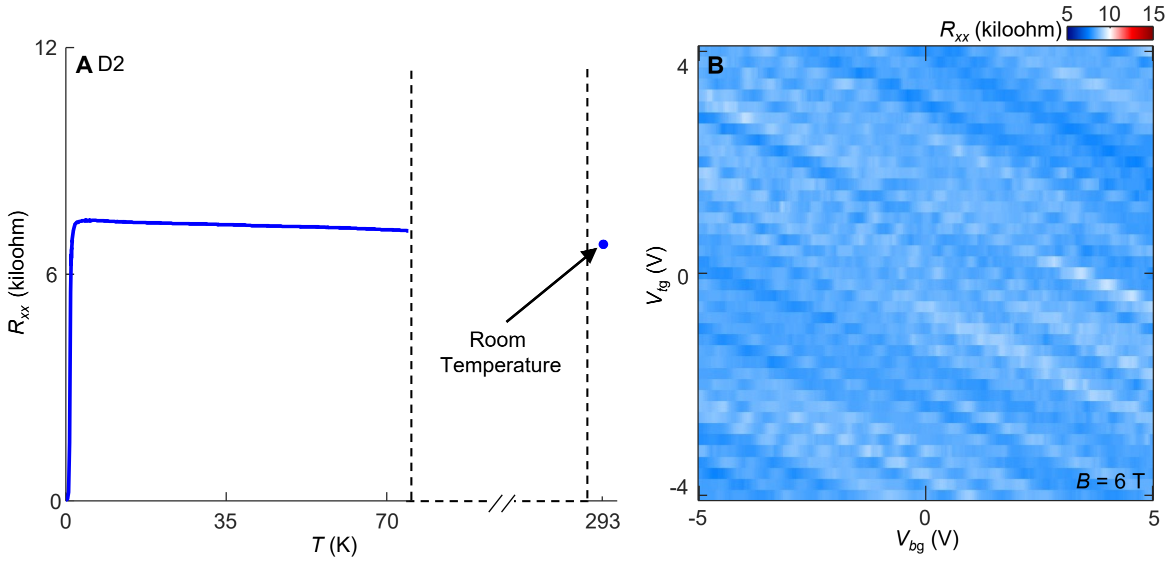

Fig. S8. Temperature- and gate-dependent normal state resistance (R_{xx}) of the moiré junction (D2). Normal state resistance of the moiré junction shows little temperature (**A**) or gate (**B**) dependence.

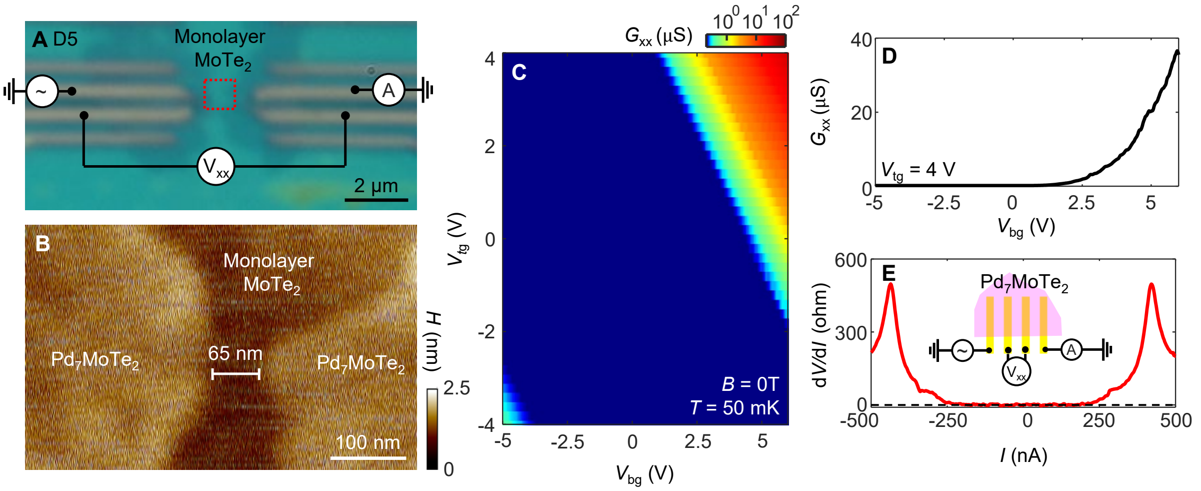

Fig. S9. Absence of residual conduction and superconductivity in a monolayer $MoTe_2$ junction. A, Optical image of device D5 with monolayer 2H-$MoTe_2$ after Pd growth, with the measurement configuration for data in **C** & **D** indicated. The location of junction is highlighted by a red box. **B,** An AFM image of the monolayer junction, revealing a width of ~ 65 nm. **C,** Dual-gate-dependent conductance ($G_{xx} = V_{xx}/I_{ac}$) map of the junction taken at 50 mK and zero magnetic field, where strong band insulator state is observed. I_{ac} is the applied low frequency ac current. **D.** G_{xx} as a function of bottom gate voltage (V_{bg}) while the top gate V_{tg} is fixed at 4 V. **E,** Differential resistance (dV/dI) *v.s.* applied DC current (I) for the Pd_7MoTe_2 pad, confirming its superconductivity. The measurement configuration for Pd_7MoTe_2 is indicated in the inset. The junction doesn't exhibit the anomalous residual conduction and is not proximitized.

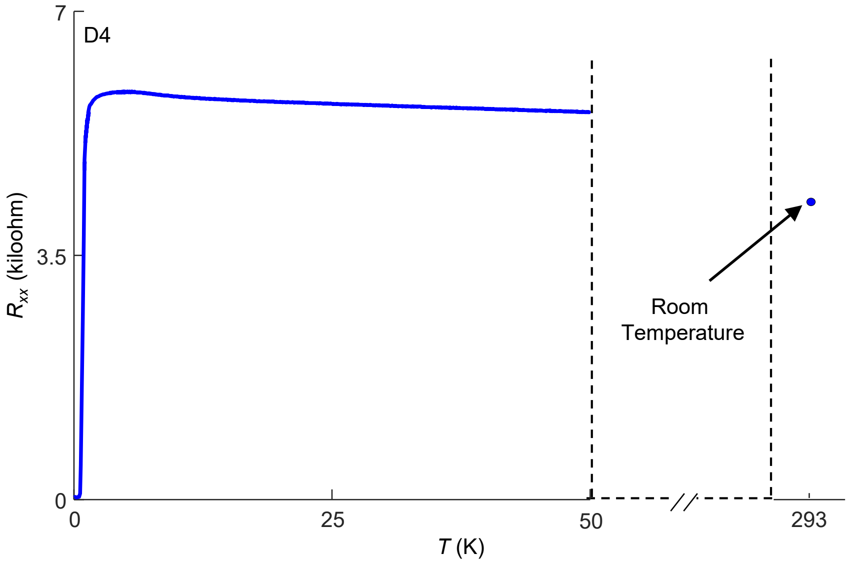

Fig. S10. Temperature-dependent R_{xx} of the natural bilayer junction (D4). The normal state resistance of the natural bilayer junction exhibits a weak temperature dependence similar to the moiré junction.

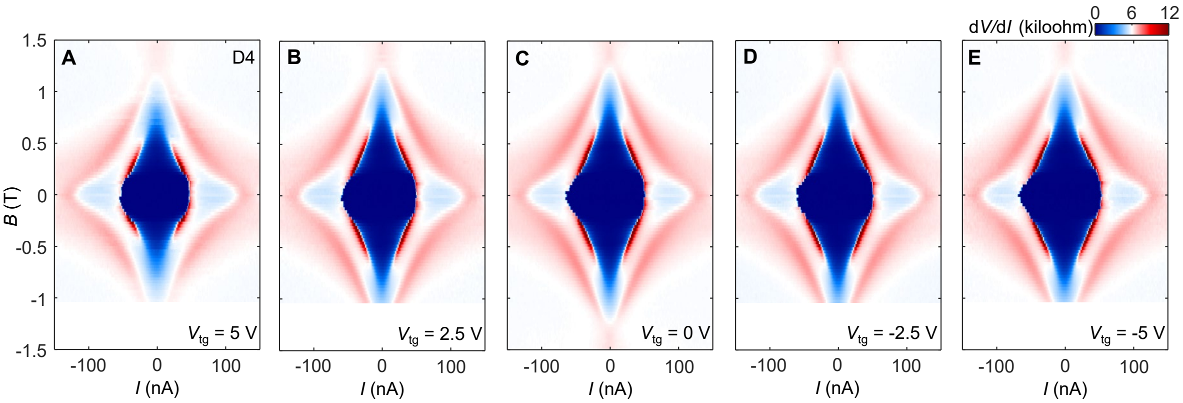

Fig. S11. The absence of the SC anomalies in natural bilayer junction at all gate voltages (D4). A-E, Magnetic field (B) dependence of dV/dI *v.s.* I map taken across the junction at various gate voltages, all confirming the absence of SC anomalies in the natural bilayer junction.

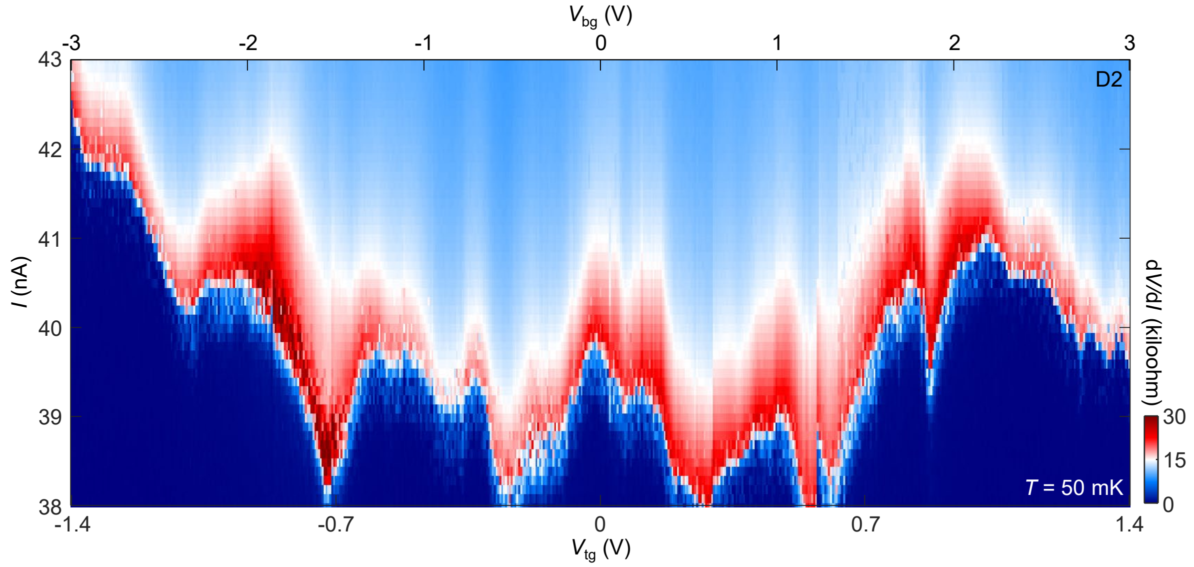

Fig. S12. Gate modulation of the junction critical current in D2. Critical current develops weak but interesting modulation in response to gate voltages. Both top (V_{tg}) and bottom (V_{bg}) gate voltages are simultaneously tuned in this experiment as indicated by bottom and top x-axes. Careful studies of the gate dependent phenomena will be a future focus.
